# Clinical Outcome of Heart Transplantation in Children and Young Adults with Congenital and Acquired Heart Disease in a Middle-Income Country: A 20-Year Experience from a Single Center in Brazil

**DOI:** 10.21470/1678-9741-2024-0420

**Published:** 2025-10-15

**Authors:** Candice Torres de Melo Bezerra Cavalcante, Valdester Cavalcante Pinto Júnior, Isabel Cristina Leite Maia, Andrea Consuelo de Oliveira Teles, Marcos Aurélio Barboza de Oliveira, T. A. Chan, CMS Schaffer, Klébia Magalhães Pereira Castello Branco

**Affiliations:** 1 Faculdade de Medicina, Universidade de Fortaleza (UNIFOR), Fortaleza, Ceará, Brazil; 2 Department of Medicine, Hospital Dr. Carlos Alberto Studart Gomes, Fortaleza, Ceará, Brazil; 3 Graduate Program in Medical Sciences, Universidade de Fortaleza (UNIFOR), Fortaleza, Ceará, Brazil; 4 Department of Pediatrics, Division of Pediatric Critical Care and the Heart Center, Seattle Children’s Hospital, University of Washington, Seattle, United States of America; 5 Department of Medicine, Universidade Federal do Ceará, Fortaleza, Ceará, Brazil

**Keywords:** Heart Transplantation, Pediatric, Heart Failure, Congenital Heart Disease, Cardiomyopathy.

## Abstract

**Introduction:**

Orthotopic heart transplantation (OHT) has become the standard of care for children with end-stage heart failure refractory to medical or surgical therapy. Despite the improvement in perioperative survival in the last decades, the long-term complications and mortality remain significant. This report examines the experience of a single center in Brazil with pediatric OHT, focusing on long-term results and mortality.

**Methods:**

This is a retrospective study from January 2002 to December 2022. Data collection consisted of demographic data, indication for heart transplantation, immunosuppression, main complications, and mortality.

**Results:**

There were 77 OHT in 74 patients. The median age at the time of OHT was 11.5 years (interquartile range 0.25 - 22 years). The indications for OHT were congenital heart disease in 46.8%, cardiomyopathy in 45.5%, and retransplantation in 3.9% of the patients. There was an average of 2.2 rejection episodes/patient and 1.3 infection episodes/patient during the first year of follow-up. The most common long-term complications were acute kidney injury (51%), systemic arterial hypertension (40.5%), and post-transplantation diabetes mellitus (10.4%). Overall survival after one year of OHT was 89.6% and fiveand 10-year survivals were 80% and 59%, respectively.

**Conclusion:**

Heart transplant is an acceptable therapeutic option for children and young adults in middle-upper income countries, with outcomes and long-term follow-up close to those of high-resource countries.

## INTRODUCTION

**Table:** 

Abbreviations, Acronyms & Symbols				
AKI	= Acute kidney injury		ESRD	= End-stage renal disease
AR	= Acute rejection		HLHS	= Hypoplastic left heart syndrome
ASD	= Atrial septal defect		IgG	= Immunoglobulin G
AZA	= Azathioprine		IQR	= Interquartile range
BID	= Twice a day		ISHLT	= International Society for Heart and Lung Transplantation Registry
BiVAD	= Biventricular assist device		IV	= Intravenous
BP	= Blood pressure		L-TGA	= Levo-transposition of the great arteries
CAV	= Cardiac allograft vasculopathy		MCS	= Mechanical circulatory support
CHD	= Congenital heart disease		MMF	= Mycophenolate mofetil
CI	= Confidence interval		MPS	= Mycophenolate sodium
CICU	= Cardiac intensive care unit		OHT	= Orthotopic heart transplantation
CKD	= Chronic kidney disease		OR	= Odds ratio
CMP	= Cardiomyopathy		PCR	= Polymerase chain reaction
CMV	= Cytomegalovirus		PO	= Orally
CPAP	= Continuous positive airway pressure		PTDM	= Post-transplantation diabetes mellitus
CPB	= Cardiopulmonary bypass		RRT	= Renal replacement therapy
CsA	= Cyclosporine		SAH	= Systemic arterial hypertension
CVD	= Cardiovascular disease		SCr	= Serum creatinine
D-TGA	= Dextro-transposition of the great arteries		Tac	= Tacrolimus
EBV	= Epstein-Barr virus		VAD	= Ventricular assist device
ECMO	= Extracorporeal membrane oxygenation		VSD	= Ventricular septal defect

Pediatric heart transplantation was first attempted in the United States of America by Adrian Kantrowitz in a 17-day-old child with severe Ebstein’s anomaly in 1967, but it was not until 1984 that Denton Cooley performed a successful pediatric heart transplantation in a child who survived 13 years after the procedure^[1^,^2]^. In Brazil, the first pediatric heart transplant was performed by Barbero-Maciel in 1992^[3]^.

Orthotopic heart transplantation (OHT) has become the standard of care for children with end-stage heart failure refractory to medical or surgical therapy, whether secondary to underlying congenital heart disease (CHD) or cardiomyopathy (CMP). Because of the shortage of pediatric donors, it is restricted to patients without other viable therapeutic options^[4^-^6]^.

According to the Twenty-fourth Pediatric Heart Transplantation Report of the International Society for Heart and Lung Transplantation Registry (ISHLT), the number of pediatric heart transplant recipients increased worldwide as heart transplantation became more widely available. Between 2010 and 2018, a total of 210 centers performing heart transplants in pediatric recipients contributed data to the Registry. Over the same time period, the proportion of pediatric heart transplants performed outside of North America and Europe increased from 3% to 7.5%^[4]^. This suggests that heart transplantation is being performed at centers outside of high-resource settings^[6^,^7]^.

In 2022, 13 pediatric centers in Brazil reported data to the Brazilian Registry of Transplants, collectively performing 32 pediatric heart transplants. This accounted for 8.9% of all heart transplants in the country, with a national pediatric heart transplant rate of 0.5 per million children. Besides that, organ donation rates in Brazil remain low, with an organ utilization rate of approximately 27%. As a result, waitlist mortality for pediatric patients remains high at 29%^[8^,^9]^.

In the Northeast of Brazil, only few centers perform heart transplants in children, and the Hospital Dr. Carlos Alberto Studart Gomes (Fortaleza, Ceará) is the only one performing heart transplants in neonates and infants. This study aims to present the 20-year experience in pediatric OHT at a single center in Northeast Brazil, with a focus on survival rates and long-term outcomes. We hypothesize that, despite the inherent challenges of transplantation in a resource-limited setting, outcomes at our center will be comparable to those reported in other national and international programs, reflecting the efficacy of our management strategies and post-transplant care.

## METHODS

We carried out a retrospective study on 74 patients who had undergone OHT at a tertiary center in the Northeast region of Brazil (Fortaleza, Ceará), from January 2002 to December 2022. This study was reported according to the Strengthening the Reporting of Observational Studies in Epidemiology (or STROBE) guidelines^[10]^. The study was approved by the Ethics in Research Committee of our institution (CAAE 18631813500005039).

A careful review of the medical records was performed to collect demographic, clinical, and surgical data about the patient population. Additional data from the preoperative period, such as the use of mechanical ventilation, vasoactive medications, and mechanical circulatory support (MCS), was also collected. Data from follow-up included: immunosuppression, incidence rate of rejection and infection, cardiac allograft vasculopathy (CAV), lymphoproliferative disease, acute kidney injury (AKI), systemic arterial hypertension (SAH), post-transplantation diabetes mellitus (PTDM), graft dysfunction, indications for retransplantation, and mortality.

### Surgical Techniques

Organ harvesting followed a protocol that optimally addressed the needs of each recipient. Standard strategies included myocardial protection by means of cardioplegia using hypothermia (4 to 8 °C) and crystalloid HTK Custodiol® solution, with an infusion time of six minutes and volume between 30 and 50 ml/kg. Sutured labels were used for identification of the anterior surface of vessels and the inlet tract of right and left pulmonary veins. As dictated by the anatomy of the recipient, the heart was harvested with various portions of the adjoining vessels.

Cardiopulmonary bypass in the recipient was performed with hypothermia (28 °C) and cannulation of the aorta, superior vena cava, and right femoral vein. Cannulation of the brachiocephalic trunk or left femoral artery and superior vena cava were performed as necessary for individual anatomic variations. Generally, the unipulmonary-bicaval anastomosis technique was employed with variations as necessary to adapt to the recipient’s underlying anatomy.

### Immunosuppression

The immunosuppression protocol of our institution is summarized at Table 1.

**Table 1:** Immunosuppression protocol in pediatric heart transplantation.

Phase	Medication	Dosage and Administration	Notes
Induction	Methylprednisolone	20 - 25 mg/kg, IV, every 12 h for 2 days (starting after CPB)	-
	Anti-thymocyte globulin	0.5 mg/kg/dose, IV, for 7 - 10 days	-
Maintenance	Calcineurin inhibitor (tacrolimus [preferred] or cyclosporine)	Tacrolimus: 0.15 - 0.3 mg/kg/day, PO, divided doses (12 h)	Tacrolimus is the drug of choice
		Cyclosporine: continuous IV (0.1 - 0.2 mg/kg/h) → PO (10 - 20 mg/kg/day), divided every 8 - 12 h	
	Antiproliferative agent (azathioprine → MMF/MPS)	Azathioprine (3 mg/kg/day, PO) was replaced by MMF/MPS (125 - 500 mg, every 12 h, PO)	MMF/MPS is now standard
	Corticosteroids	Used in high-risk rejection patients	Discontinued if no rejection occurs within 6 months
Rejection treatment	Methylprednisolone bolus therapy	20 - 25 mg/kg, BID, for 4 days	-
	Prednisone	Continued after first rejection episode	Discontinued if no recurrence after 6 months
	T-cell antibodies	Used for persistent rejection or rejection with ventricular dysfunction	-
Infection prophylaxis	Ganciclovir (CMV)	For CMV-IgG negative recipients with CMV-IgG positive donors	-
	Toxoplasmosis prophylaxis	For IgG-negative recipients with IgG-positive donors	-

BID=twice a day; CMV=cytomegalovirus; CPB=cardiopulmonary bypass; IgG=immunoglobulin G; IV=intravenous; MMF=mycophenolate mofetil; MPS=mycophenolate sodium; PO=orally

### Follow-up

Routine follow-up was conducted by a multidisciplinary team through scheduled consultations per protocol (once a week for the first month, every 15 days in the second and third months, once a month until one year, and every 1 - 2 months for lifetime). Clinical evaluation consisted of echocardiography, electrocardiogram, routine laboratory tests (complete blood count and platelets, basic electrolytes, renal and hepatic function, triglycerides, cholesterol, glucose, and pro-B-type natriuretic peptide), and immunosuppressive trough levels where appropriate. Testing for toxoplasmosis (immunoglobulin G [IgG] and immunoglobulin M) titers and polymerase chain reaction (PCR) for cytomegalovirus and Epstein-Barr virus (EBV) occurred every 3 - 6 months.

Acute rejection (AR) episodes in infants and children were primarily diagnosed based on echocardiographic findings and clinical presentation, with endomyocardial biopsy performed only when necessary. Older children and adolescents had the diagnosis of rejection mainly through endomyocardial biopsy.

Clinical signs of graft rejection and echocardiographic findings are described below:

• Echocardiographic findings: ventricular dysfunction, increase thickening of septal and left ventricular posterior wall, new atrioventricular regurgitation, pericardial effusion.
• Signs of heart failure: Rales, hepatosplenomegaly, oliguria, tachypnea, progressive global cardiomegaly, pulmonary edema, and/or pleural effusion.
• Nonspecific symptoms: Irritability, malaise, and changes in feeding or sleeping patterns.

We chose to base our diagnosis and rejection treatment on non-invasive methods in infants and young children because of a greater than expected complication rate, such as tricuspid valve injury and perforation, in this population. It might also be important to mention that, at the beginning of the program, we did not have a pediatric hemodynamic specialist.

The majority of patients underwent routine biopsies on an annual basis. Serious infection was defined as one requiring hospitalization, intravenous antibiotics, or both. Graft coronary vasculopathy was annually assessed by coronary angiography after the first year of transplantation. To monitor for post-transplant lymphoproliferative disorder, EBV PCR was collected every three months, and computed tomography was requested if there was any suspicion of malignancy.

AKI definitions were based on the Kidney Disease Improving Outcomes (or KDIGO) criteria. Stage 1 AKI was defined as a > 50% or 0.3 mg/dl (within 48 hours) increase in serum creatinine (SCr) from preoperative values, stage 2 was a two-fold increase in SCr, and stage 3 was a three-fold increase in SCr or requiring dialysis during hospitalization^[11]^. Severe AKI was defined as stage 2 or 3 AKI.

SAH was defined as the sustained elevation of blood pressure (BP) above normal levels, generally considered as a systemic BP of ≥ 130 mmHg or a diastolic BP of ≥ 80 mmHg in adolescent, and a BP above 95th percentile for age, sex, and height in pediatric patients^[12]^. PTDM was defined by persistent post-transplant hyperglycemia in clinically stable patients^[13]^.

### Statistical Analysis

Continuous variables are summarized as median and interquartile range (IQR). Categorical variables are summarized by frequency with percentage. Continuous variables were compared between cohorts using the unpaired Student’s *t*-test for means of normally distributed continuous variables or the Wilcoxon rank sum tests for skewed data. The χ2 or Fisher’s exact tests were used to compare differences in proportions among the categorical data. We compared the results between survivors and non-survivors, using one-year post-transplant survivor to separate the groups. Survival was assessed using the Kaplan-Meier estimator.

Cumulative survival curves were constructed according to the Kaplan-Meier methods. To assess the association between clinical conditions, diagnosis, and age with post-transplant survival, a Cox proportional regression model was constructed. A *P* < 0.05 was considered significant. Data were analyzed with IBM SPSS Statistics for Windows, version 20 (IBM Corp., Armonk, N.Y., USA).

## RESULTS

### Patient Population

We identified 74 patients who underwent cardiac transplantation during the 20-year study period (Table 2). The age of the patients ranged from three months to 22 years (median 11.5 years, IQR: three months - 22 years). Two patients (2.7%) were under one year of age, 30 (39%) were between one and ten years, and 42 (54.5%) were older than 10 years of age at the time of heart transplantation. Four patients older than 18 years with CHD were included in our series. There was a predominance of male patients (59.7%). The majority of patients lived in the capital of the state (46%), and 14 (18.9%) were from other states.

**Table 2:** Baseline clinical characteristics prior to transplantation.

Characteristic	N (%)	Non-survivor (N = 24)	Survivor (N = 50)	*P*-value
Sex				
Male	43 (58.1%)	13 (54.2%	14 (45.8%)	0.52
Female	31 (41.9%)	31 (62%)	19 (38%)	
Location prior to transplant				
Home	43 (58.1%)	12 (50%)	32 (64%)	0.51
Ward	6 (8.1%)	0	5 (10%)	
CICU	25 (33.7%)	12 (50%)	13 (26%)	
Geographic region of residence				
Capital city	33 (44.6%)	14 (58.3%)	19 (31%)	
Outside of capital city	27 (36.5%)	4 (16.7%)	23 (46%)	
Other state	14 (18.9%)	6 (25%)	8 (16%)	
Use of mechanical ventilation	8 (10.8%)	4 (16.7%)	4 (8%)	0.26
Use of CPAP	2 (2.7%)	2 (8.3%)	0 (0%)	0.03
Use of vasoactive drugs	27 (36.5%)	12 (46.2%)	14 (28%)	0.063
AKI requiring renal replacement therapy	8 (10.8%)	7 (29.2%)	1 (2%)	0.0001
Mechanical circulatory support	3 (4%)	1 (4.2%)	2 (4%)	0.98

AKI=acute kidney injury; CICU=cardiac intensive care unit; CPAP=continuous positive airway pressure

Thirty-five (45.5%) patients were transplanted due to CMP, and 36 (46.8%) due to CHD (Table 3). Among those with CHD, 25 (69.4%) patients had single-ventricle physiology, and 30 patients (83.3%) had undergone previous surgery prior to OHT.

**Table 3:** Indication for heart transplantation.

Indications for OHT	N (%)	Non-survivor (N = 24)	Survivor (N = 50)	*P*-value
Congenital heart disease	36 (46.7%)	12 (50%)	24 (48%)	0.85
Glenn	11 (30.6%)			
Univentricular^*^	6 (16.6%)			
Fontan	2 (5.5%)			
Ebstein	2 (5.5%)			
L-TGA	3 (8.4%)			
D-TGA + VSD + pulmonary stenosis	1 (2.8%)			
HLHS	6 (16.6%)			
Tetralogy of Fallot	3 (8.4%)			
ASD + VSD	1 (2.8%)			
Others	1 (2.8%)			
Cardiomyopathy	35 (45.4%)	11 (45.8%)	24 (48%)	
Complete heart block^**^	2 (2.6%)	0 (0%)	1 (2%)	
Rheumatic heart disease	1 (1.3%)	1 (4.2%)	1 (2%)	
Retransplant	3 (4%)	2 (66.6%)	1 (33.4%)	

* Single ventricle without surgery;

** Complete heart block with congenital heart disease or cardiomyopathy

ASD=atrial septal defect; D-TGA=dextro-transposition of the great arteries; HLHS=hypoplastic left heart syndrome; L-TGA=levo-transposition of the great arteries; OHT=orthotopic heart transplantation; VSD=ventricular septal defect

We performed a combined heart-kidney transplantation in a 15-year-old adolescent with complex CHD with univentricular physiology and chronic kidney disease (CKD). He had never undergone any previous surgical intervention, nor had he been under regular follow-up care. After seven days on the transplant waiting list, he underwent heart transplantation, and the kidney transplant was performed 19 hours later using organs from the same donor. From a cardiac standpoint, the postoperative period was uneventful, with no cardiac complications. However, the patient continued to require hemodialysis for 20 days following the kidney transplant. He was discharged from hospital 50 days after the procedures.

### Number of Transplantation

**Fig. 1:**
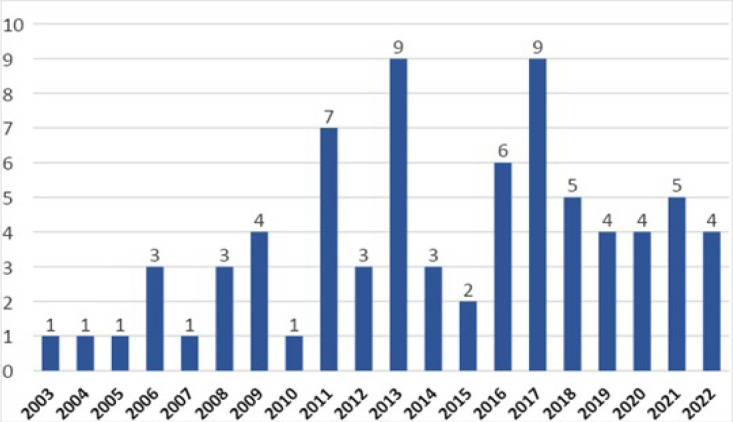
Number of pediatric heart transplants per year.

The average number of OHT per year for the entire period was 3.8 transplants/year. Over the most recent decade, there has been an upward trend in the number of transplants (average = 5.7 OHT/year) (Figure 1).

### Circulatory Support

The MCS program at our center began in 2012 and has been used to support patients in both pre and postoperative settings. Among our cohort, three patients (3.9%) with dilated CMP received MCS prior to OHT. One patient was supported with extracorporeal membrane oxygenation (ECMO) for 12 days before transplantation but unfortunately died eight hours after the procedure due to primary graft dysfunction. Another patient received a biventricular assist device (BiVAD) - Centrimag® - 48 hours prior to OHT and experienced an uneventful postoperative course. This BiVAD was made available through donation. The third patient was supported with ECMO for 16 days and recovered without complications, being discharged home 50 days after transplantation. In Brazil, the use of durable BiVAD systems remains extremely limited due to high costs and restricted access. Most centers, including ours, do not have routine availability of this technology, making its use an exceptional circumstance. This limitation reflects broader challenges faced by middle-income countries and may impact the outcomes of patients requiring advanced mechanical support.

ECMO was used in three patients with severe graft failure after OHT. Two patients were supported for 72 hours with recovery of cardiac function and subsequently discharged from the hospital on postoperative days 43 and 26. Another patient received ECMO support 48 hours after OHT but developed renal failure requiring renal replacement therapy (RRT) and ultimately died 96 hours after OHT. Two patients presented late with severe acute antibody mediated rejection and cardiogenic shock 10 and four years after the transplant, respectively. The first patient was supported for nine days but died from multiple organ dysfunction syndrome. The other patient used ECMO for 11 days, with partial recovery of cardiac function, survived and was discharged from hospital four months later due to post fungal infection.

### Immunosuppressive Therapy

**Fig. 2:**
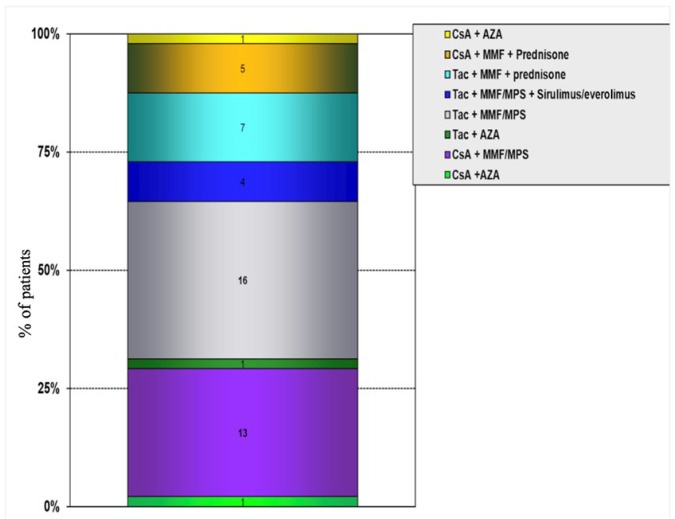
Immunosuppressive therapy. AZA=azathioprine; CsA=cyclosporine; MMF=mycophenolate mofetil; MPS=mycophenolate sodium; Tac=tacrolimus.

The majority of patients receive tacrolimus (58.3%) and mycophenolate (93.7%) for immunosuppression. Twenty patients are on cyclosporine (41.6%). The main immunosuppressive regimens are presented in Figure 2.

### Follow-up and Complications

Infectious and rejection episodes were the most common complications during follow-up. The majority of patients (82%) experienced at least one episode of AR throughout the follow-up period, with 70% (52 patients) having at least one AR episode within the first year post-transplant. Over the 20 years of follow-up, the average number of rejection episodes per patient was 2.2 (IQR 1 - 4). All infections requiring treatment were documented, with an overall infection rate of 2.3 episodes per patient. Notably, we observed only one death from infection in our cohort, a 10-year-old child with influenza infection, eight months after transplantation. The most common long-term complications were AKI (51%), SAH (40.5%), and PTDM (10.4%).

### Graft Vascular Disease and Lymphoproliferative Disease

There were three cases (2.6%) of cardiovascular disease (CVD) and one case (1.2%) of lymphoproliferative disease during the study period. Among the three patients with CVD, two patients underwent retransplantation, and the other one died on the waiting list. Lymphoproliferative disease was detected in a 12-year-old boy 5.3 years after transplant, who subsequently died during the treatment.

### Renal Dysfunction

We identified eight patients with severe renal dysfunction prior to transplantation. Among them, one patient underwent a combined heart-kidney transplant with a good renal function within one month postoperatively. Over the 20-year study period, the post-transplant mortality rate for patients with preoperative renal failure requiring RRT was 87.5%, and for those not requiring RRT was 30.4% (*P* < 0.001). Multivariate logistic regression analysis demonstrated that the need for RRT before OHT was independently associated with higher post-transplant mortality (adjusted odds ratio [OR]: 16.0; 95% confidence interval [CI]: 1.8 - 138).

After transplant, 51.3% (38/74) of patients experienced at least one episode of transient AKI. Additionally, two patients progressed to CKD and ultimately developed end-stage renal disease (ESRD), requiring RRT. Both patients died due to rejection at 6.5 and seven years post-transplantation.

### Retransplants

The retransplantation rate in our series was 4%. A total of three (3.9%) patients were listed for retransplantation due to CAV at two, 4.5, and six years after first transplant (mean of 4.6 years). Two of these patients had CHD, and one had a diagnosis of CMP. The overall survival after retransplantation was 33.3% (one patient experienced sudden death two years and nine months after the retransplantation, and the other died due to multiple organ failure in the early postoperative period).

### Overall survival

During the 20-year follow-up, the overall mortality was 33.8% (26 patients), and the causes of death are listed in Table 2. When we compared patients who underwent OHT in the first decade with those in the second decade of our program, there was an increase in survival (mortality 48.6% *vs.* 22.2%, *P* = 0.01). The median interval between transplantation and death was 3.8 years (IQR 0 - 6.9 years). The 30-day survival was 95.9% (71/74). There were eight deaths within the first year of follow-up (early mortality). The causes of early deaths were: one intraoperative death due to graft failure before the beginning of MCS program and seven patients (three receiving MCS) died early in the postoperative period (< 72 hours) due to multiple organ dysfunction and graft dysfunction. All other deaths occurred beyond one year after OHT, with a median interval of 6.4 years (IQR 3.6 - 7.4 years) between transplantation and death. Notably, these late deaths predominantly occurred in adolescents and were associated with non-adherence to immunosuppressive therapy. The median follow-up was 5.7 years (IQR 3.7 - 8.5 years), conditioned to one-year survival. The survival rate at one, five, and ten years were 89.6%, 80%, and 59%.

**Fig. 3:**
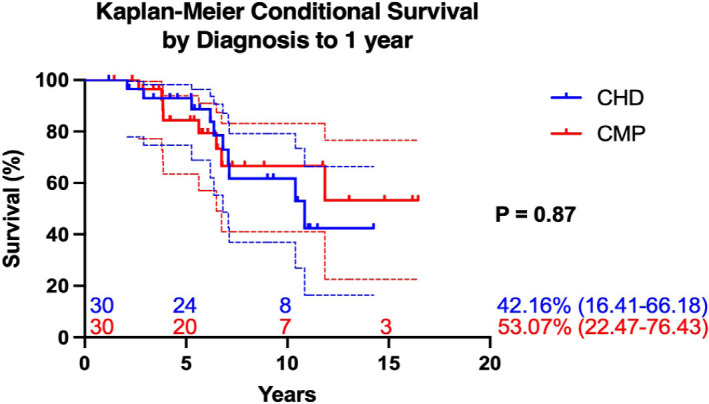
Kaplan-Meier conditional survival by diagnosis to 1 year. CHD=congenital heart disease; CMP=cardiomyopathy.

In this population, there was no difference in overall survival between patients with a pre-transplant diagnosis of CHD and CMP (mean 9.7 ± 0.9 years and 11 ± 1.2 years, respectively; *P* = 0.97). Furthermore, when survival was conditioned to the first year post-transplant, we still observed no significant difference between the two groups (mean 10.6 ± 0.91 and 12.45 ± 1.24 years, respectively; *P* = 0.66) (Figure 3).

**Fig. 4:**
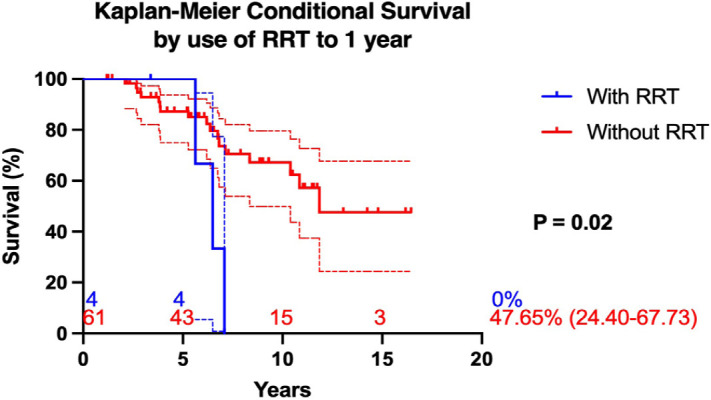
Kaplan-Meier conditional survival by use of RRT to 1 year. RRT=renal replacement therapy.

Patients who required RRT before undergoing OHT had significantly lower post-transplant survival compared to those with normal pre-transplant renal function (5.41 ± 0.63 *vs.* 13 ± 0.91, *P* = 0.0001). The overall mortality rate in the RRT group was 87.5%, whereas patients with preoperative renal dysfunction who did not require RRT had a lower but still considerable mortality rate of 30.4% (*P* < 0.001). Multivariate logistic regression analysis confirmed that the need for RRT before transplantation was an independent predictor of increased post-transplant mortality (adjusted OR: 16.0; 95% CI: 1.8 - 138) (Figure 4).

## DISCUSSION

Although the first successful pediatric transplant in Brazil occurred in 1992, the first transplant in Northeastern Brazil was only possible 10 years later, supported by the Adult Heart Transplant Program of our own institution and the Pediatric Heart Transplant Program of the Instituto do Coração (InCor) of São Paulo. Here we report our 20-year single-center experience in heart transplantation in children and young adults with congenital and acquired heart disease.

Our institution is a referral center for pediatric cardiac surgery and heart transplantation to the North and Northeast regions of Brazil. Early and late mortalities have significantly improved during recent decades in pediatric patients after OHT. Reasons for this advance in the outcomes include better selection of patients, improved donor organ preservation, modifications in surgical technique, and advances in perioperative care. However, several factors have continued to negatively impact post-transplant survival, including graft failure, AR, coronary vasculopathy, infection, malignancy, and renal failure^[4^-^7^,^14]^.

### Infections and Rejections

Rejection episodes and infections were the most common complications during our follow-up period. Rejection remains one of the primary post-transplant complications, and while it is more frequent in the first year after transplantation, it can occur at any time^[15^-^17]^. Over the 20 years of follow-up, we observed an average of 2.3 ± 2.1 rejection episodes per patient, and 70% (52/74) of patients had at least one episode of AR in the first year after transplantation. We observed an incidence of rejection five times higher than that reported to the ISHLT^[5]^. Some factors that could contribute to this higher incidence would be a non-invasive diagnosis in many cases, which may have overestimated the diagnosis, a greater proportion of adolescents (potentially a risk-factor for non-adherence to treatment), and a higher percentage of patients with diagnosis of CHD, commonly associated with previous surgery and blood transfusion with higher allosensitization. However, it is important to note that we did not have comprehensive data on human leukocyte antigen (or HLA) mismatch, panel-reactive antibody levels, or donor-specific antibodies for all patients, particularly in the earlier era cases of our program. This lack of data represents a limitation, as these factors could provide valuable insights and could help in future comparisons.

Overall, the incidence of AR between discharge and one year after transplant has declined over time, and the rate was 13.3% between 2012 and 2018, with lower incidence among recipients discharged on tacrolimus during this same period.

The absence of routine angiographic evaluation in the early years of our program, when a pediatric interventional cardiologist was not available to perform examinations in young children, may have contributed to the lower reported rates of CAV diagnosis compared to centers in high-income countries. However, our findings are consistent with those of other transplant centers in Brazil^[6^,^18]^.

Infection remains an important cause of morbidity and mortality, accounting for approximately 12% of deaths during the first year following transplantation according to the Registry of 2017 and varying from 5.2% to 6.2% after one year of transplant^[7]^. Our incidence rate of infections was 2.3 episodes/patient, which was slightly higher when compared to centers in well-resourced countries, potentially due to the lower socioeconomic level of our population. Nonetheless, we had only one death due to infection^[7^,^18]^.

### Renal Dysfunction

In our series, we had eight patients (10.8%) with severe renal dysfunction before transplant, and these patients had higher long-term mortality. Previous studies have demonstrated that pre-existing renal insufficiency is common and varies from 2.5 to 42% depending on the definition used. Pre-transplant renal insufficiency has a direct correlation with postoperative renal dysfunction and is associated with earlyand long-term mortality^[19^-^24]^. In the Twenty-fourth Pediatric Heart Transplantation Report by the ISHLT, the incidence of the use of RRT before transplant was 3.3% between 2010 and 2018, and it was associated with lower survival within 12 months after discharge (90.8% *vs.* 72.9%, *P* < 0.01)^[5]^.

Renal dysfunction is a common source of morbidity in the post-transplant period, with most patients experiencing at least mild renal impairment^[20^,^25^-^27]^. This dysfunction is typically multifactorial. Contributing factors include pre-transplant renal function, perioperative hemodynamic instability, graft function, rejection episodes, dehydration, and infections. However, long-term nephrotoxicity from immunosuppressive drugs, particularly calcineurin inhibitors, remains a major contributing factor^[19^-^25]^.

In our cohort, pre-transplant renal dysfunction significantly impacted on post-transplant outcomes. Patients requiring RRT before transplantation had an exceptionally high post-transplant mortality rate of 87.5%, whereas those with renal dysfunction not requiring RRT had a much lower mortality rate of 30.4% (*P* < 0.001). Multivariate analysis confirmed the need for pre-transplant RRT as an independent risk factor for post-transplant mortality (adjusted OR: 16.0; 95% CI: 1.8 - 138). Additionally, more than half (51.3%) of our patients experienced at least one episode of post-transplant transient AKI, and two progressed to ESRD, ultimately dying from rejection at 6.5 and seven years after transplantation.

The Twenty-Fourth Pediatric Heart Transplantation Report (2021) of the ISHLT indicates that the need for RRT post-transplant ranges from 6% to 17%, depending on age group^[7]^. Many single-center studies have reported variable progression of renal dysfunction over time, with a progressively higher risk of severe impairment in patients with lower baseline glomerular filtration rates. Within 10 years post-transplant, 3 - 10% of pediatric heart transplant recipients develop severe renal dysfunction^[19^-^27]^. Our findings underscore the critical importance of a multidisciplinary team, including a nephrologist in the care of patients in the pre-transplant period, particularly for patients with advanced renal dysfunction, as well as the need for long-term vigilant monitoring to mitigate renal complications and improve post-transplant survival.

### Retransplantation

The overall retransplant rate has increased over the last few decades, rising from 0.5% between 2001 and 2009 to 2.7% between 2010 and 2018^[5]^. However, retransplantation is a significant risk factor for oneand five-year mortality^[5]^. We had three cases (4%) of retransplantation over the last 20 years due to graft dysfunction with a survival rate of 33.3%. Coronary vasculopathy as an indication for retransplantation and longer inter-transplant interval were associated with better survival after retransplantation, but overall survival remains lower than for primary transplantation^[6]^. Feingold et al.^[28]^ published a study in pediatric OHT with more than 4,000 cases showing increased mortality due to retransplantation, although some authors have found no difference compared to patients undergoing primary OHT^[26^-^31]^. We had a similar incidence of retransplantation, but the low number of cases makes comparison between groups impaired.

### Circulatory Support

Due to shortage of donor organs in the pediatric group, MCS is now routinely utilized to provide short-term and long-term support as a bridging strategy to increase survival on the waiting list for OHT, reaching values > 50% in patients older than one year with dilated CMP and 11.8 to 20.1% in patients with CHD (including ECMO, ventricular assist device [VAD], and total artificial heart)^[6^,^7^,^32^-^34]^.

We had three cases (5.6%) of MCS as a bridge to OHT: two patients were transplanted (one in ECMO and one using BIVAD) and one died while waiting for transplant (ECMO support). Only one patient was discharged after OHT (overall survival 33.3%). Two patients supported by ECMO died: one before transplantation and another eight hours post-transplant. Although MCS use has increased survival on the pediatric heart transplantation waiting list, post-transplant survival for patients bridged to transplant with ECMO has continued to be associated with worse survival; however, other forms of MCS (VADs) resulted in survival similar to that of patients not on MCS^[6^,^7^,^32^-^37]^.

In our state, financial constraints often prevent the acquisition of VADs, resulting in a significantly lower number of patients receiving MCS compared to data reported by the ISHLT Registry^[6^,^7]^. In our cohort, MCS was also utilized in the postoperative period of OHT: two patients required ECMO support due to early graft dysfunction, and two others were supported during episodes of AR with severe ventricular dysfunction. These limitations highlight the urgent need to expand access to MCS as a bridge to transplantation in Brazil. Strategies to reduce waitlist mortality should include earlier referral for VAD therapy, increased awareness and promotion of organ donation, and better resource allocation for pediatric cardiac care. Moreover, multicenter studies are essential to assess national trends in pediatric heart transplantation and MCS use, identify regional disparities, and develop solutions aimed at improving equity and outcomes across the country.

### Early and Late Mortality

Despite the lower number of transplants, we had a high early survival rate (94.4%). The overall survival is influenced by the volume of transplants performed in the centers, according to the ISHLT, with the larger mortality in smaller centers (< 4 pediatric heart transplants performed per year). Our early mortality was similar to the data from national and international studies^[5^,^18^,^38^,^39]^.

In contrast to the findings of the Twenty-fourth Pediatric Heart Transplantation Report (2021), which demonstrated better survival in patients under 10 years of age and those with a primary diagnosis of CMP, our study did not reveal a statistically significant difference in survival between patients diagnosed with CMP and those with CHD. This finding may be due to the small sample size, limiting statistical power^[5]^.

Our analysis indicated that patients with pre-transplant AKI requiring RRT exhibited significantly higher post-transplant mortality compared to those without renal dysfunction. This finding aligns with previous studies, such as the one by Zafar et al.^[35]^, which demonstrated that pediatric heart transplant recipients requiring perioperative RRT had decreased survival rates at 30 days, and one, five, and 10 years post-transplant. These results underscore the critical importance of early nephrology intervention and meticulous management of renal function in pediatric patients awaiting heart transplantation^[19]^.

### Limitations

This paper has some limitations: (1) this is a single-center study with a small number of patients; (2) some data from early patients were not available; and (3) endomyocardial biopsy data for diagnosing rejection in younger children may have influenced the reported rejection rates.

## CONCLUSION

Heart transplant is an acceptable therapeutic option for children and young adults, even in small centers with outcomes comparable to large centers. While there may be challenges in resource-limited settings, it is still possible to achieve good long-term outcomes for children with life-threatening heart disease. Certainly, there is a lot of room for improvement, such as infrastructure in MCS/VAD, technology, and multidisciplinary care, especially in early nephrology referral in pre-transplant CKD.

## Data Availability

The authors declare that the data supporting the findings of this study are available within the article.

## Artificial Intelligence Usage

The authors declare use of ChatGPT for terminology translation. The content produced by the artificial intelligence tool was revised and edited by the authors as necessary, and they take full responsibility for the content to be published.
